# Associations between non-insulin-based insulin resistance indices and diabetic nephropathy in patients with diabetes mellitus in US adults: a cross-sectional study of NHANES 1999–2018

**DOI:** 10.3389/fendo.2024.1458521

**Published:** 2024-12-10

**Authors:** Fan Zhang, Yan Han, Yonghua Mao, Wenjian Li

**Affiliations:** ^1^ Changzhou Clinical College, Xuzhou Medical University, Changzhou, China; ^2^ Department of Endocrinology, Changzhou Third People’s Hospital, Changzhou, China; ^3^ Department of Clinical Nutrition, Changzhou Third People’s Hospital, Changzhou, China; ^4^ Department of Urology, Changzhou Third People’s Hospital, Changzhou, China

**Keywords:** insulin resistance, non-insulin-based, diabetic nephropathy, diabetes mellitus, NHANES

## Abstract

**Objective:**

This study investigated the associations between non-insulin-based insulin resistance indices (METS-IR, TyG, TG/HDL, and TyG-BMI) and the risk of diabetic nephropathy (DN) in US adults with diabetes mellitus (DM).

**Methods:**

This study was based on the 1999-2018 National Health and Nutrition Examination Survey (NHANES) database and included 6,891 patients with DM for cross-sectional analysis. Multivariate adjusted models and restricted cubic spline (RCS) models were employed to assess the association between the insulin resistance index and the risk of DN. Subgroup analyses were conducted to explore the impact of different population characteristics.

**Results:**

The results indicated that higher quartiles of METS-IR, TyG, TG/HDL, and TyG-BMI were associated with a significantly increased risk of DN. After adjusting for multiple covariates, including gender, age, and race, the associations between these indices and the risk of DN remained significant, with corresponding odds ratios (ORs) of 1.51 (95% confidence interval [CI]: 1.29-1.76), 2.06 (95% CI: 1.77-2.40), 1.61 (95% CI: 1.38-1.88), and 1.57 (95% CI: 1.35-1.84), with all P-values less than 0.001. RCS analysis indicated a nonlinear relationship between these indices and the risk of DN. The TyG index exhibited a highly consistent association with the risk of DN in all models.

**Conclusion:**

Non-insulin-based insulin resistance indices are significantly associated with the risk of DN. The TyG index is a superior tool for assessing the risk of DN. These indices can assist in identifying patients at risk of DN, thereby enabling the implementation of more effective preventive and therapeutic strategies.

## 1 Introduction

Diabetes mellitus (DM), a prevalent metabolic disease with a worrisome global epidemic, is a significant public health concern (1). It is projected that the total number of individuals with diabetes worldwide will reach 780 million by 2045, a figure that represents a substantial threat to human health and well-being. Concurrently, the global prevalence of kidney disease is considerable, affecting approximately 850 million individuals. Chronic kidney disease (CKD) represents the predominant form of kidney disease, with a global prevalence of 9.1% (2). Although the onset and progression of CKD are influenced by various factors, including impaired fasting glucose, hypertension, high body mass index (BMI), a high-sodium diet, and a high-lead diet, DM is undoubtedly one of the most significant contributing factors (2). It is noteworthy that approximately 40% of patients with DM develop diabetic nephropathy (DN), which represents the most common and severe complication of DM (3–6). The principal clinical manifestations of DN include a significant reduction in glomerular filtration rate (GFR), abnormally elevated urinary albumin levels, and symptoms of hypertension. These pathophysiologic changes may eventually lead to end-stage renal disease (ESRD) (3, 7–9). Statistical analysis indicates that patients with DN exhibit a markedly elevated risk of all-cause mortality, reaching up to approximately 30 times that of diabetic patients without DN (10). This underscores the significant role of DN as a contributor to diabetes-related mortality (11). Consequently, it is paramount to identify and clarify the risk factors associated with DN to prevent its occurrence, delay its progression, and improve the quality of life of those affected.

Insulin resistance (IR) is defined as a reduction in cellular sensitivity to insulin, which results in a decline in the effectiveness of insulin in facilitating glucose uptake and utilization. Further research has demonstrated that insulin resistance plays a central role in the pathogenesis of diabetes and that its association with DN is also receiving increasing attention (12–14). Specifically, insulin resistance contributes to DN’s progression through various biological mechanisms, including exacerbating renal hemodynamic disturbances, impairing podocyte function, inhibiting normal tubular function, and promoting glomerular hypertrophy and tubulointerstitial fibrosis (15, 16). Furthermore, several clinical studies have demonstrated that the severity of insulin resistance is strongly associated with increased microalbuminuria and significantly reduced glomerular filtration rate (eGFR) in diabetic patients (17–19). These findings collectively indicate that insulin resistance plays a pivotal role in the pathogenesis of DN and represents a critical link in the complex chain of this disease.

The hyperinsulin-normoglycemic clamp method (HEC) is the gold standard for assessing IR. However, despite its status as the gold standard, the HEC has not gained widespread acceptance in practical applications due to its high cost and complex procedure (20, 21). Furthermore, the homeostasis model assessment of insulin resistance (HOMA-IR) index, another frequently utilized method for assessing IR, presents similar challenges (20, 22). The high cost of plasma insulin or C-peptide measurements, coupled with the need for more standardization in clinical practice, has constrained the adoption of the HOMA-IR index. This is particularly the case for diabetic patients, as most of them are treated with insulin, making accurate measurement of insulin difficult, thus compromising the accuracy of the HOMA-IR index (22). Moreover, the HOMA-IR cannot reflect the intricate dynamic relationship between glucose and insulin metabolism. This is because it is based on a single point in time and is therefore unable to capture the dynamic changes in the glucose-insulin feedback system fully (23). Consequently, developing more efficient, economical, and accurate IR assessment methods is significant for clinical practice and scientific research.

To more accurately assess and manage IR in diabetic patients, researchers have developed a series of non-insulin-based IR indices, such as the metabolic insulin resistance score (METS-IR), the triglyceride-glucose (TyG), triglyceride-to-high-density lipoprotein cholesterol ratio (TG/HDL-C), and the triglyceride-glucose body mass index (TyG-BMI), etc. METS-IR is an emerging method for assessing IR with the added benefit of evaluating an individual’s cardiometabolic risk (24, 25). It is calculated based on a series of standardized measurements, including fasting plasma glucose (FPG), triglycerides (TG), high-density lipoprotein cholesterol (HDL-C), and BMI. Studies have demonstrated that METS-IR is as effective as the classic HOMA-IR index in assessing IR levels, and in some cases, it outperforms it (26). The TyG index, another innovative index for IR assessment, combines triglyceride and FBG levels and has the potential to serve as a reliable biomarker for IR (27). Notably, the TyG index not only possesses higher sensitivity than traditional homeostasis models but has also been confirmed by several studies to be independently and significantly associated with the risk of DN in individuals with decreased renal function (28), especially in individuals with type 2 diabetes mellitus (T2DM) (29, 30). Furthermore, the ability of the TyG index to predict DN is even better than that of the HOMA-IR index (29, 31). Moreover, a high TyG index has been demonstrated to be positively correlated with the risk of ESRD, further underscoring its pivotal role in predicting renal complications in diabetes (14). TG/HDL-C has garnered considerable attention as a straightforward predictor of IR. Previous studies have demonstrated that this ratio is not only strongly associated with IR status but also positively correlated with diabetes risk (32, 33). The ability of the TG/HDL-C ratio to predict the onset of diabetes is particularly significant when the ratio exceeds 0.35 (34). Finally, TyG-BMI, as a complement and extension of TyG, also demonstrated a high degree of correlation with IR, providing an additional reliable option for IR assessment (35).

In the current field of research on non-insulin-based IR indices and the risk of DN in patients with DM, although there is a wealth of research on the association between the TyG index and DN, there is a lack of in-depth exploration of the relationship between the METS-IR, TG/HDL, and TyG-BMI and DN. Furthermore, the majority of these studies have focused on Asian populations. In light of the limitations above, the primary objective of this study was to investigate the potential association between non-insulin-based insulin resistance indices and the development of DN among diabetic patients in the context of the U.S. population. This study aims to employ a big data-driven analytic strategy to clearly define and validate the efficacy and value of different IR indices in predicting and assessing the risk of DN. Furthermore, to construct a more comprehensive understanding framework, this study will examine the intricate interactions between these IR indices and potential influencing factors, including age, gender, demographic characteristics, lifestyle habits, and coexisting chronic diseases. This will facilitate the elucidation of the multidimensional mechanisms of IR in developing DN.

## 2 Materials and methods

### 2.1 Research participants

All data for this study were obtained from the 1999-2018 National Health and Nutrition Examination Survey (NHANES) database. This database contains the results of cross-sectional surveys conducted every two years by the Centers for Disease Control and Prevention (CDC). The research protocol of the NHANES project strictly followed the guidelines of the Ethics Review Committee of the National Center for Health Statistics (NCHS). It ensured that all participants signed an informed consent form. Furthermore, during the data analysis phase, NIH policy regulations were followed. Given the anonymity and non-direct contact nature of the data, it was used directly in the study without needing additional ethical review. The study adhered rigorously to the standards set forth by the Strengthening the Reporting of Observational Studies in Epidemiology (STROBE) initiative, ensuring the highest quality in study design and reporting.

**Figure 1:**
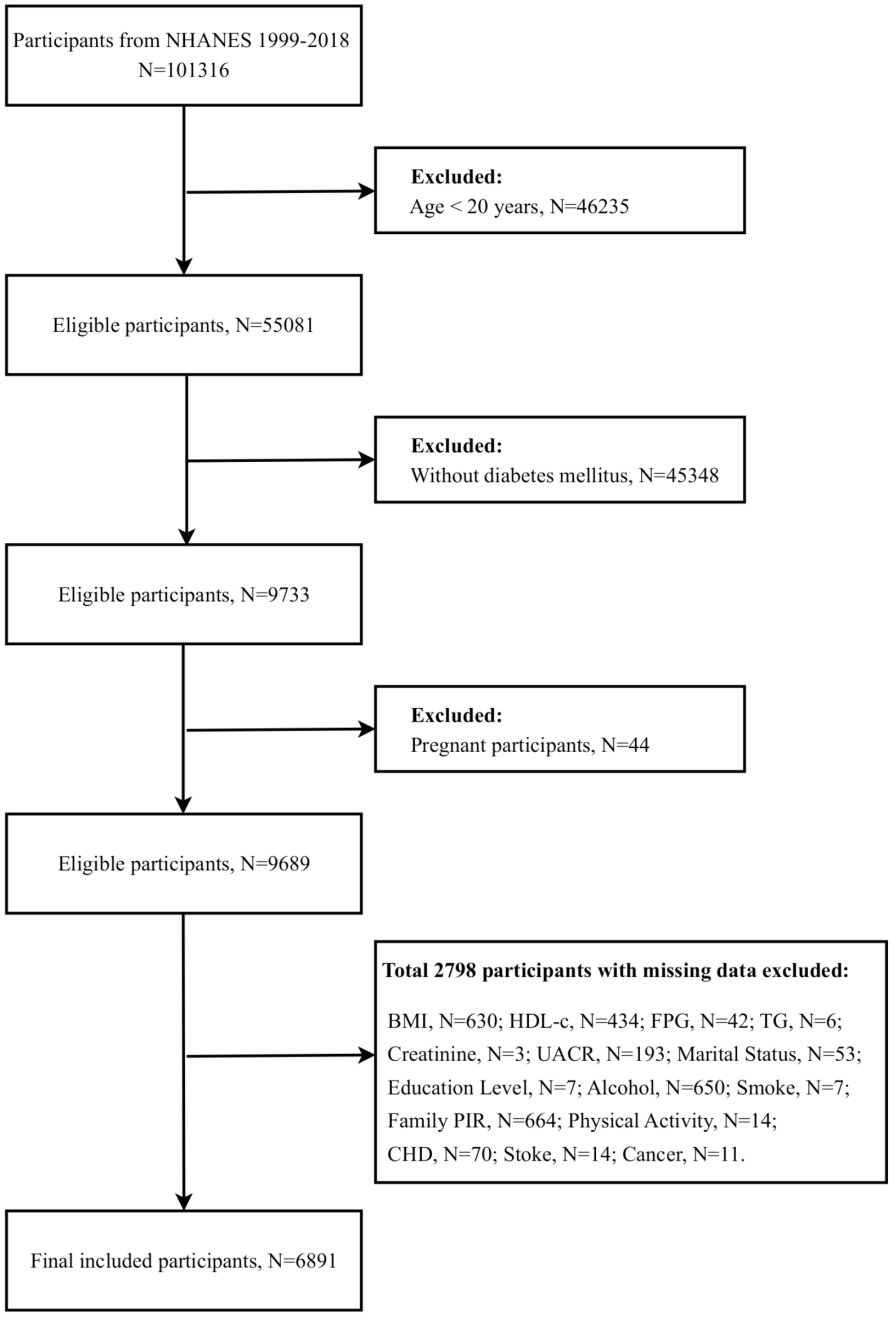
Participant screening flowchart. BMI, Body mass index; HDL-c, High density lipoprotein cholesterol; FPG, Fasting plasma-glucose; TG, Triglyceride; UACR, Urinary albumin/creatinine ratio; PIR, Poverty-to-income ratio; CHD, Coronary heart disease.

At the study’s outset, a sample population was drawn from ten consecutive survey cycles, resulting in 101,316 participants. To ensure the accuracy and relevance of the study results, we implemented a rigorous data cleaning and exclusion process to exclude ineligible participants. These exclusions included individuals under the age of 20, non-diabetic patients, pregnant females, and those with missing data, particularly on demographic characteristics, chronic disease status, biomarkers related to IR, and diagnostic indicators of DN. Following the implementation of a rigorous screening process, 6,891 eligible participants were identified for analysis in this study (**Figure 1**).

### 2.2 Definition of disease

The following criteria were employed to define DM in this study: (1) a precise diagnosis by a healthcare professional, (2) FPG at or above the threshold of 126 mg/dl, (3) glycosylated hemoglobin (HbA1c) level of not less than 6.5%, and (4) the individual was receiving diabetic medication or insulin therapy. We employed two core indicators to assess renal function: the urine albumin-to-creatinine ratio (UACR) and the eGFR. The eGFR was calculated according to the recommended formula by the Collaborative Group on Epidemiology of Chronic Kidney Disease (CKD-EPI). To diagnose DN, we employed the internationally recognized criteria, which stipulate that a UACR value of not less than 30 mg/g or an eGFR value of less than 60 mL/min/1.73 m^2^ must be met.

### 2.3 Assessment of the non-insulin-based IR indices

To ensure the accuracy and reliability of the results, we employ the following scientifically validated formulas in the assessment of IR:

METS-IR is calculated by the formula Ln[2 × FPG(mg/dl) + TG(mg/dl)] × BMI(kg/m²)/Ln[HDL-C(mg/dl)] (24). TyG is calculated by the formula Ln[TG(mg/dl) × FPG(mg/dl)/2] (27). TG/HDL-C is calculated by dividing the TG (mg/dL) by the HDL-C (mg/dL) (36). TyG-BMI is calculated by the formula TyG ×BMI(kg/m²) (35).

All biochemical measurements were conducted after a minimum of 8.5 hours of fasting, utilizing an automated biochemical analyzer to guarantee the precision of the data. FPG, TG, and HDL-C concentrations were measured in strict accordance with standard operating procedures. Meanwhile, BMI was calculated as a standardized body mass indicator by dividing weight (kg) by the square of height (m).

### 2.4 Covariate assessment

To ascertain the association between the IR Index and DN, we constructed multivariate adjustment models to resolve the potential impact of confounding variables on this relationship. The covariates included in this study were gender, age, race, education, marital status, household economic status, alcohol intake, smoking behavior, physical activity level, and a history of a range of important chronic diseases, including hypertension, coronary heart disease (CHD), stroke, and cancer. Race was classified as Mexican American, Non-Hispanic White, Non-Hispanic Black, and Other Race. The sample was divided into three educational attainment categories based on the years of education completed: less than 9th grade, 9th through 12th grade, and more than 12th grade. Marital status was simplified into two categories: cohabitation and solitude. This was done to explore the role of family structure factors. To categorize household economic status, income was carefully divided into three intervals based on the Poverty-to-Income Ratio (PIR) criterion, as officially defined by the U.S. government. These intervals were designated as low (PIR ≤1.3), medium (PIR > 1.3 to ≤3.5), and high (PIR > 3.5). This study assessed smoking and drinking habits using standardized assessment methods. Smoking status was defined based on whether the participant had smoked more than 100 cigarettes in their lifetime and whether they were a current smoker. Alcohol consumption was assessed by asking whether the participant had consumed at least 12 alcoholic beverages of any type in the past year. Physical activity was classified into three categories: vigorous, moderate, and inactive. A comprehensive medical history was obtained for each participant, encompassing hypertension, CHD, stroke, and cancer. For hypertension, participants were queried as to whether they had ever been informed by a medical professional that they had hypertension or were currently taking medication for it. For CHD, participants were asked whether they had ever been diagnosed with the condition, whether they had experienced angina or a heart attack, or whether they were currently undergoing treatment for it. Similarly, participants were asked whether they had ever been informed by a medical professional that they had experienced a stroke. Finally, participants were queried as to whether they had ever been diagnosed with cancer.

### 2.5 Statistical analysis

For continuous variables, the Shapiro-Wilk test was employed to verify the normality of the data. Based on the test results, the mean ± standard deviation or median (25th and 75th percentile) was selected to characterize the variables according to their normal distribution. One-way analysis of variance (ANOVA) or Kruskal-Wallis nonparametric tests were employed to assess the existence of statistically significant differences between groups concerning the distribution characteristics of the variables in question. Categorical variables were presented as frequencies and percentages, and the chi-square test was employed to analyze differences between groups.

To gain insight into the intricate relationship between IR indices and DN, we constructed logistic regression models to assess the impact of each index and its quartiles on the risk of DN. This was accomplished by estimating the ratio of ratios (ORs) and their 95% confidence intervals (CIs). Three levels of multivariate-adjusted models were gradually built to eliminate the potential interference of confounding variables. Model 1 served as the baseline without any adjustment. Model 2 incorporated essential demographic characteristics such as age, gender, and race. Model 3 further introduced educational attainment, marital status, family PIR, smoking and drinking habits, level of physical activity, and history of chronic diseases such as hypertension, CHD, stroke, and cancer as adjustment variables to enhance the explanatory power and predictive accuracy of the model.

To ascertain the existence of a potential nonlinear dose-response relationship between the IR indices and DN, a restricted cubic spline (RCS) model was employed. In this model, the IR indices were considered a continuous variable. Based on their distributional properties, the 5th, 35th, 65th, and 95th percentiles were selected as critical points for analysis. Should a nonlinear association be observed, a likelihood ratio test was employed to ascertain the critical point or threshold effect between the indices and the risk of DN with greater precision.

Furthermore, subgroup analyses were conducted to stratify the participants based on variables such as gender, education, marital status, family PIR, smoking and drinking habits, and the presence of hypertension, CHD, stroke, and cancer. This was done to explore the heterogeneity of the pattern of the association between IR index and DN among subgroups with different characteristics. Through interaction analysis, we evaluated the stability and consistency of the association between IR index and DN risk within each subgroup.

Throughout the statistical analysis, the principle of a two-sided test was followed, and a p-value of less than 0.05 was considered statistically significant. All data analysis was conducted using the R 4.4.0 software (provided by the R Foundation at http://www.R-project.org) in conjunction with the SPSS version 23.0 (IBM Corporation, Armonk, New York, USA) statistical package. Graphical presentations were generated using GraphPad Prism version 9.0 (GraphPad Software, USA).

## 3 Results

### 3.1 Baseline characteristics

In this study, the baseline characteristics of 6,891 patients with DM were analyzed. Of these, 2,660 were diagnosed with DN, and 4,231 were not. The results of the statistical analysis indicated that, although there was no significant difference in the distribution of gender between the two groups (*p* = 0.183), there were statistically significant differences in the age structure, ethnic composition, education level, marital status, and family economic status (all *p* < 0.05). In particular, the DN patient population exhibited a higher mean age, reaching 67 years, compared to a mean age of 60 for non-DN patients. Non-Hispanic white and black individuals comprised a significantly higher percentage of DN patients compared to other racial groups. Regarding educational attainment, a more significant proportion of patients with DN had lower levels of education. The analysis of marital status revealed a significantly higher proportion of patients with DN living alone. In contrast, analysis of family economic status, as measured by the PIR, showed that low income was more concentrated among individuals with DN. Further analysis of lifestyle and health status revealed significant differences between DN and non-DN patients in terms of smoking, drinking habits, physical activity participation, and the prevalence of multiple chronic diseases. The proportion of smokers was higher in the group of DN patients, whereas the proportion of alcohol consumers and those with a high level of physical activity were relatively lower. Moreover, the prevalence of hypertension, CHD, stroke, and cancer was significantly higher in patients with DN, underscoring the complexity of the association between these diseases. At the biochemical level, significant differences were observed in FPG, HbA1c, total cholesterol (TC), TG, UACR, and eGFR between patients with and without DN. These differences directly reflected the impaired renal function and metabolic abnormalities observed in patients with DN. Notably, BMI, HDL-C, and specific IR indices such as METS-IR and TyG-BMI did not show significant differences between the two groups (**Table 1**).

**Table 1:** Baseline characteristics of participants with diabetes mellitus.

Variables	Total (n = 6891)	Non-DN (n = 4231)	DN (n = 2660)	*P*
Gender, n (%)				0.183
Male	3679 (53.39)	2232 (52.75)	1447 (54.40)	
Female	3212 (46.61)	1999 (47.25)	1213 (45.60)	
Age (years)	62.00 (51.00, 71.00)	60.00 (48.00, 67.00)	67.00 (58.00, 76.00)	<0.001
Race, n (%)				<0.001
Mexican American	1386 (20.11)	874 (20.66)	512 (19.25)	
Non-Hispanic White	2665 (38.67)	1547 (36.56)	1118 (42.03)	
Non-Hispanic Black	1633 (23.70)	998 (23.59)	635 (23.87)	
Other Race	1207 (17.52)	812 (19.19)	395 (14.85)	
Education Level, n (%)				<0.001
Less than 9th grade	1245 (18.07)	684 (16.17)	561 (21.09)	
9–12th grade	1170 (16.98)	675 (15.95)	495 (18.61)	
More than 12th grade	4476 (64.95)	2872 (67.88)	1604 (60.30)	
Marital Status, n (%)				<0.001
Cohabitation	4170 (60.51)	2694 (63.67)	1476 (55.49)	
Solitude	2721 (39.49)	1537 (36.33)	1184 (44.51)	
Family PIR, n (%)				<0.001
Low (≤1.3)	2407 (34.93)	1407 (33.25)	1000 (37.59)	
Medium (1.3–3.5)	2785 (40.42)	1654 (39.09)	1131 (42.52)	
High (*>*3.5)	1699 (24.66)	1170 (27.65)	529 (19.89)	
Smoke, n (%)				0.001
Yes	3532 (51.26)	2104 (49.73)	1428 (53.68)	
No	3359 (48.74)	2127 (50.27)	1232 (46.32)	
Alcohol, n (%)				<0.001
Yes	4170 (60.51)	2635 (62.28)	1535 (57.71)	
No	2721 (39.49)	1596 (37.72)	1125 (42.29)	
Physical Activity, n (%)				<0.001
Inactive	3266 (47.40)	1814 (42.87)	1452 (54.59)	
Moderate	2233 (32.40)	1426 (33.70)	807 (30.34)	
Vigorous	1392 (20.20)	991 (23.42)	401 (15.08)	
Hypertension, n (%)				<0.001
Yes	4302 (62.44)	2374 (56.11)	1928 (72.51)	
No	2588 (37.56)	1857 (43.89)	731 (27.49)	
Coronary heart disease, n (%)				<0.001
Yes	675 (9.80)	292 (6.90)	383 (14.40)	
No	6216 (90.20)	3939 (93.10)	2277 (85.60)	
Stroke, n (%)				<0.001
Yes	522 (7.58)	217 (5.13)	305 (11.47)	
No	6369 (92.42)	4014 (94.87)	2355 (88.53)	
Cancer, n (%)				<0.001
Yes	953 (13.83)	512 (12.10)	441 (16.58)	
No	5938 (86.17)	3719 (87.90)	2219 (83.42)	
BMI (kg/m^2^)	30.82 (26.97, 35.97)	30.90 (27.10, 36.03)	30.70 (26.83, 35.87)	0.168
FPG (mg/dL)	131.00 (108.00, 168.00)	129.00 (107.00, 158.00)	136.00 (110.00, 188.00)	<0.001
HbA1c (%)	6.70 (6.00, 7.80)	6.60 (5.90, 7.50)	6.90 (6.20, 8.20)	<0.001
TC (mg/dL)	185.00 (157.00, 217.00)	187.00 (159.00, 217.00)	181.50 (153.00, 218.00)	0.002
TG (mg/dL)	155.00 (105.00, 233.00)	151.00 (103.00, 225.00)	163.00 (108.00, 246.00)	<0.001
HDL-c (mg/dL)	45.00 (38.00, 55.00)	45.00 (39.00, 55.00)	45.00 (38.00, 55.00)	0.215
Creatinine (mg/dL)	0.90 (0.72, 1.10)	0.82 (0.70, 0.97)	1.09 (0.82, 1.36)	<0.001
UACR (mg/g)	12.40 (6.50, 37.53)	8.26 (5.42, 13.73)	59.55 (27.54, 176.01)	<0.001
eGFR (ml/min/1.73m^2^)	85.83 (66.53, 100.84)	92.09 (79.05, 104.17)	62.20 (48.87, 91.47)	<0.001
METS-IR	49.98 (42.10, 59.52)	49.97 (42.04, 59.46)	49.99 (42.28, 59.75)	0.519
TyG	9.24 (8.76, 9.80)	9.18 (8.72, 9.72)	9.33 (8.82, 9.91)	<0.001
TG/HDL	3.37 (2.04, 5.78)	3.24 (1.97, 5.55)	3.64 (2.15, 6.09)	<0.001
TyG-BMI	288.55 (247.46, 339.98)	288.62 (246.88, 337.89)	288.50 (248.42, 343.01)	0.296

Data are shown as median (25th, 75th percentiles) or percentages, *p <*0.05 considered statistically signiﬁcant.

DN, Diabetic nephropathy; PIR, Poverty-to-income ratio; BMI, Body mass index; FPG, Fasting plasma-glucose; HbA1c, Hemoglobin A1c; TC, Total cholesterol; TG, Triglyceride; HDL-c, High-density lipoprotein cholesterol; UACR, Urinary albumin/creatinine ratio; eGFR, Estimated glomerular filtration rate; METS-IR, Metabolic Score for Insulin Resistance; TyG, Triglyceride-glucose; TG/HDL, Triglyceride/High-density lipoprotein; TyG-BMI, Triglyceride glucose - body mass index.

### 3.2 Relationships between IR indices and DN

To investigate the relationships between METS-IR, TyG, TG/HDL, TyG-BMI, and DN among diabetic patients, three analytic models were constructed to assess potential confounding effects comprehensively. The specific model setup was as follows: Model 1 did not include any adjustments. Model 2 incorporated gender, age, and race as adjustment variables based on Model 1. Model 3 further extended the adjustment to include educational attainment, marital status, family PIR, smoking habits, alcohol consumption status, physical activity level, and history of chronic diseases such as hypertension, CHD, stroke, and cancer. The analysis results indicated that METS-IR, TyG, TG/HDL, and TyG-BMI were significantly associated with the risk of DN. In particular, the unadjusted model demonstrated no significant association between METS-IR and DN. However, in Models 2 and 3, METS-IR demonstrated a positive correlation with the risk of DN, with the adjusted ORs remaining stable at 1.02 (95% CI: 1.01-1.02), with a p-value of <0.001. This indicates that the gender, age, and race factors significantly affect the relationship. In contrast, the TyG and TG/HDL indices demonstrated a significant association with an increased risk of DN in all models. Furthermore, the risk of DN exhibited a notable increase with increasing levels of these indices. TyG-BMI index did not demonstrate a significant association with DN in the unadjusted model; the positive association with DN risk became significant in both Model 2 and Model 3.

Further refinement of these associations through quartile analyses revealed that the high quartile groups of METS-IR, TyG, TG/HDL, and TyG-BMI were all at significantly elevated risk of DN, corresponding to ORs of 1.51 (95% CI: 1.29-1.76), 2.06 (95% CI: 1.77-2.40), 1.61 (95% CI: 1.38-1.88) and 1.57 (95% CI: 1.35-1.84), with all p-values less than 0.001. These findings strongly support the role of these IR indices as potential predictors of the development of DN in diabetic patients (**Table 2**).

**Table 2:** Relationship between METS-IR, TyG, TG/HDL, TyG-BMI, and DN in patients with diabetes mellitus in different models.

Variables	Model 1		Model 2		Model 3	
	OR (95%CI)	*P*	OR (95%CI)	*P*	OR (95%CI)	*P*
METS-IR	1.00 (1.00 ~ 1.01)	0.344	1.02 (1.01 ~ 1.02)	**<0.001**	1.01 (1.01 ~ 1.02)	**<0.001**
Categories						
Quartile 1	1.00 (Reference)		1.00 (Reference)		1.00 (Reference)	
Quartile 2	1.02 (0.89 ~ 1.17)	0.769	1.07 (0.92 ~ 1.23)	0.386	1.03 (0.89 ~ 1.20)	0.672
Quartile 3	0.99 (0.86 ~ 1.14)	0.888	1.22 (1.05 ~ 1.41)	**0.008**	1.12 (0.96 ~ 1.30)	0.136
Quartile 4	1.04 (0.91 ~ 1.19)	0.576	1.72 (1.48 ~ 2.01)	**<0.001**	1.51 (1.29 ~ 1.76)	**<0.001**
TyG	1.28 (1.20 ~ 1.36)	**<0.001**	1.50 (1.40 ~ 1.60)	**<0.001**	1.47 (1.37 ~ 1.58)	**<0.001**
Categories						
Quartile 1	1.00 (Reference)		1.00 (Reference)		1.00 (Reference)	
Quartile 2	1.03 (0.89 ~ 1.18)	0.685	1.06 (0.92 ~ 1.23)	0.432	1.06 (0.91 ~ 1.23)	0.475
Quartile 3	1.19 (1.03 ~ 1.36)	**0.016**	1.30 (1.12 ~ 1.50)	**<0.001**	1.25 (1.07 ~ 1.45)	**0.004**
Quartile 4	1.60 (1.39 ~ 1.83)	**<0.001**	2.13 (1.83 ~ 2.48)	**<0.001**	2.06 (1.77 ~ 2.40)	**<0.001**
TG/HDL	1.01 (1.01 ~ 1.02)	**0.027**	1.02 (1.02 ~ 1.03)	**<0.001**	1.02 (1.01 ~ 1.03)	**<0.001**
Categories						
Quartile 1	1.00 (Reference)		1.00 (Reference)		1.00 (Reference)	
Quartile 2	1.10 (0.95 ~ 1.26)	0.194	1.13 (0.98 ~ 1.31)	0.092	1.10 (0.95 ~ 1.28)	0.200
Quartile 3	1.23 (1.07 ~ 1.41)	**0.004**	1.38 (1.19 ~ 1.60)	**<0.001**	1.27 (1.09 ~ 1.48)	**0.002**
Quartile 4	1.34 (1.17 ~ 1.54)	**<0.001**	1.75 (1.51 ~ 2.04)	**<0.001**	1.61 (1.38 ~ 1.88)	**<0.001**
TyG-BMI	1.00 (1.00 ~ 1.00)	0.177	1.01 (1.01 ~ 1.01)	**<0.001**	1.01 (1.01 ~ 1.01)	**<0.001**
Categories						
Quartile 1	1.00 (Reference)		1.00 (Reference)		1.00 (Reference)	
Quartile 2	1.05 (0.92 ~ 1.21)	0.454	1.08 (0.93 ~ 1.24)	0.322	1.04 (0.90 ~ 1.21)	0.592
Quartile 3	0.95 (0.82 ~ 1.09)	0.439	1.16 (1.01 ~ 1.35)	**0.042**	1.08 (0.93 ~ 1.26)	0.294
Quartile 4	1.11 (0.96 ~ 1.27)	0.153	1.79 (1.54 ~ 2.09)	**<0.001**	1.57 (1.35 ~ 1.84)	**<0.001**

The bold values indicated statistically significant.

Model 1: crude.

Model 2: adjusted for Gender, Age, Race.

Model 3: adjusted for Gender, Age, Race, Education Level, Marital Status, Family PIR, Smoke, Alcohol, Physical Activity, Hypertension, Coronary heart disease, Stroke, Cancer.

DN, Diabetic nephropathy; METS-IR, Metabolic Score for Insulin Resistance; TyG, Triglyceride-glucose; TG/HDL, Triglyceride/High-density lipoprotein; TyG-BMI, Triglyceride glucose - body mass index; OR, Odds ratio; CI, Confidence interval.

**Figure 2:**
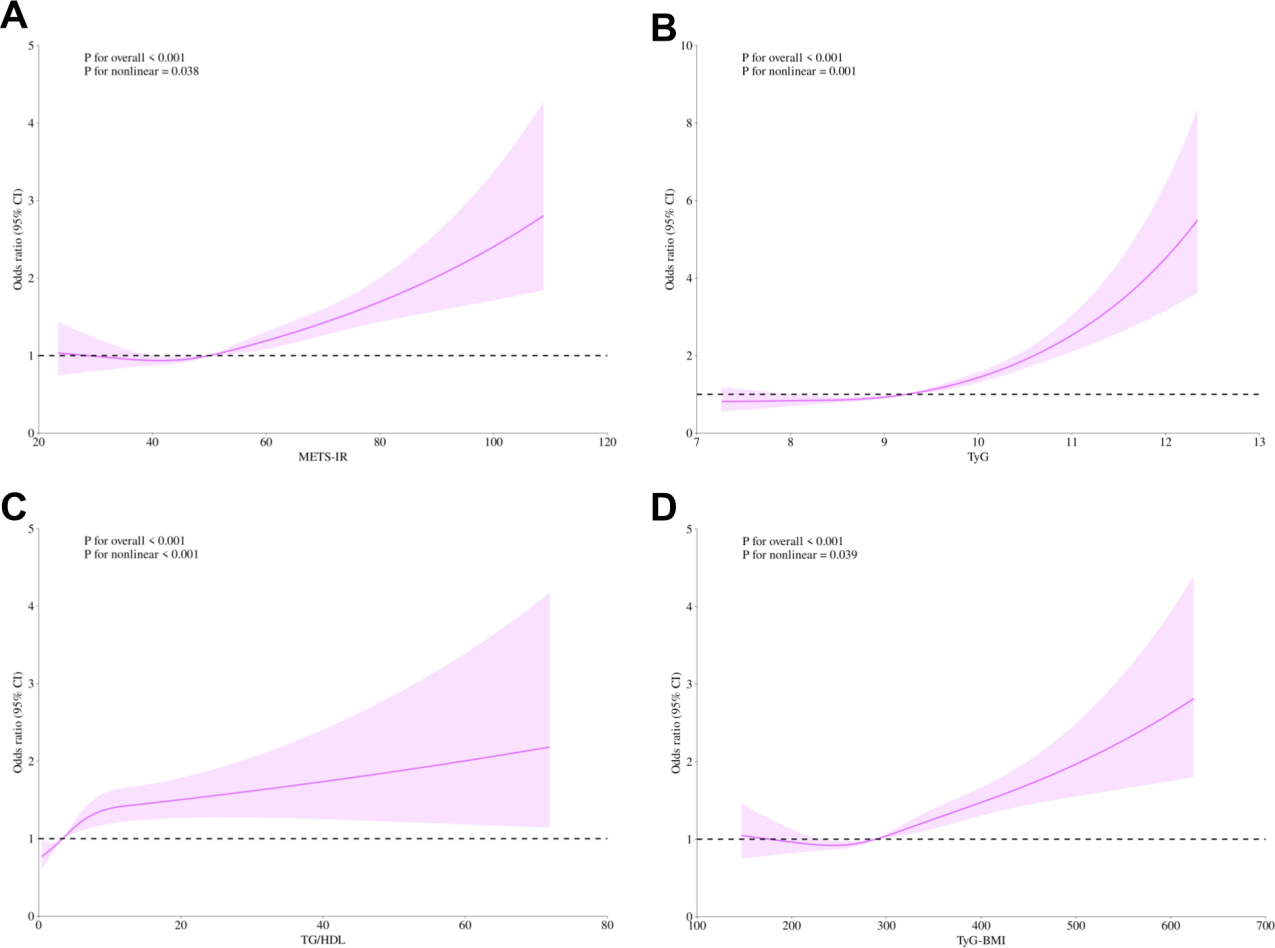
Non-linear relationship of METS-IR **(A)**, TyG **(B)**, TG/HDL **(C)**, TyG-BMI **(D)**, and diabetic nephropathy. The solid purple line displays the odds ratio, with the 95% confidence intervals represented by purple shading. They were adjusted for gender, age, race, education level, marital status, family PIR, smoking, alcohol, physical activity, hypertension, coronary heart disease, stroke, and cancer. METS-IR, Metabolic Score for Insulin Resistance; TyG, Triglyceride-glucose; TG/HDL, Triglyceride/High-density lipoprotein; TyG-BMI, Triglyceride glucose - body mass index; CI, Confidence interval; PIR, Poverty-to-income ratio.

To investigate the nonlinear relationship between the non-insulin-based IR indices and the risk of DN in diabetic patients, we employed RCS modeling. After adjusting for several potential confounding variables, including gender, age, race, education, marital status, family PIR, smoking habits, drinking status, physical activity level, hypertension, CHD, stroke, and cancer, The analyses revealed that the four IR indices (METS-IR, TyG, TG/HDL, and TyG-BMI) were not only highly significant overall correlations with DN risk (all *p*-values for overall < 0.001) but also exhibited an evident nonlinear character (*p*-values for nonlinear 0.038, < 0.001, 0.001, 0.039, respectively). Further threshold analyses were conducted to define inflection point values for each IR indices. The following values were identified: 49.98 for METS-IR, 9.24 for TyG, 3.37 for TG/HDL, and 288.55 for TyG-BMI. This finding is of particular significance, as it indicates that when IR indices exceed these critical thresholds, the risk of DN increases significantly as the index levels are further elevated (**Figure 2**).

### 3.3 Subgroup analysis

**Figure 3:**
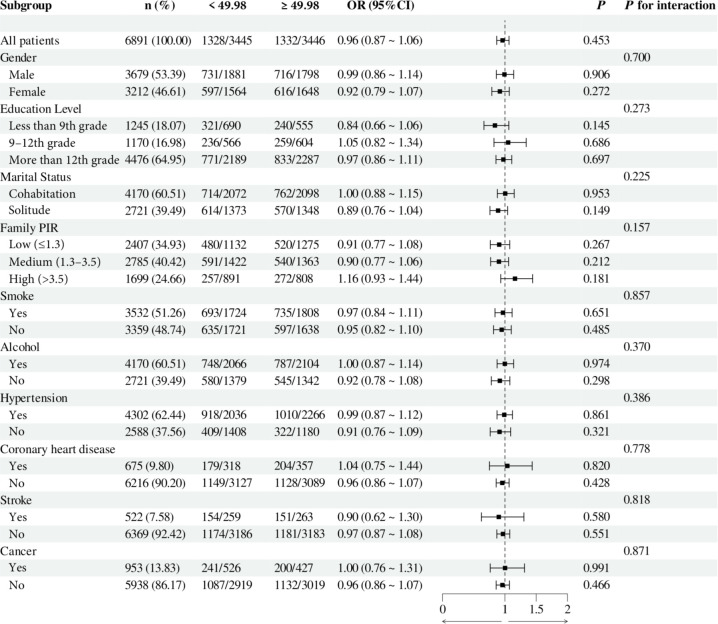
Subgroup analysis of the relationship between METS-IR and diabetic nephropathy. Adjusted variables: gender, age, race, education level, marital status, family PIR, smoking, alcohol, physical activity, hypertension, coronary heart disease, stroke, and cancer. The model was not adjusted for the stratification variables themselves in the corresponding stratification analysis. METS-IR, Metabolic Score for Insulin Resistance; PIR, Poverty-to-income ratio; OR, odds ratio; CI, confidence interval.

**Figure 4:**
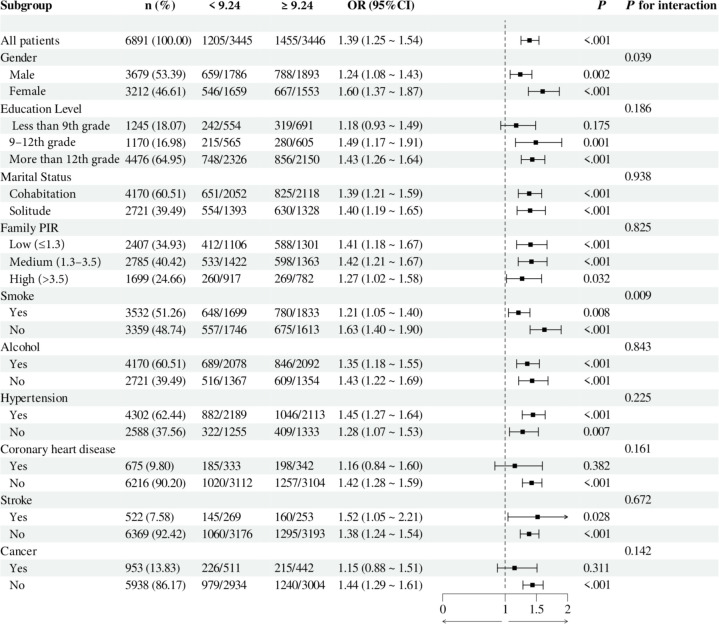
Subgroup analysis of the relationship between TyG and diabetic nephropathy. Adjusted variables: gender, age, race, education level, marital status, family PIR, smoking, alcohol, physical activity, hypertension, coronary heart disease, stroke, and cancer. The model was not adjusted for the stratification variables themselves in the corresponding stratification analysis. TyG, Triglyceride-glucose; PIR, Poverty-to-income ratio; OR, odds ratio; CI, confidence interval.

**Figure 5:**
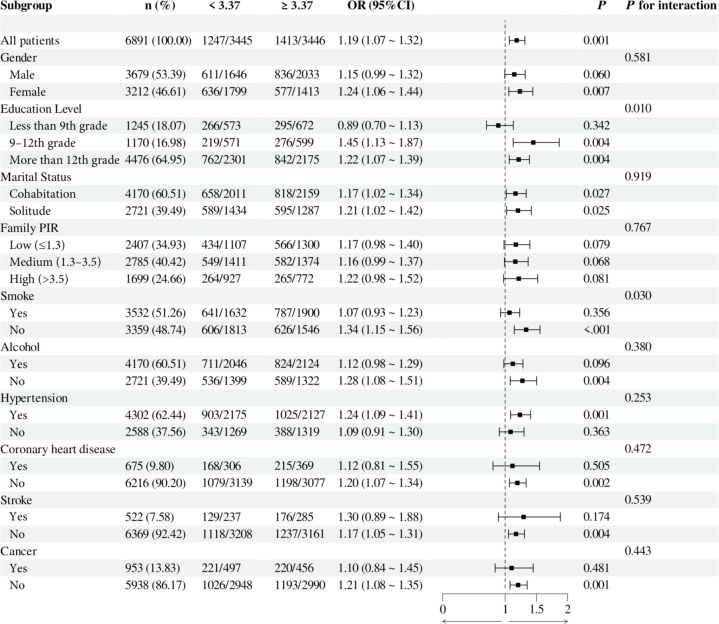
Subgroup analysis of the relationship between TG/HDL and diabetic nephropathy. Adjusted variables: gender, age, race, education level, marital status, family PIR, smoking, alcohol, physical activity, hypertension, coronary heart disease, stroke, and cancer. The model was not adjusted for the stratification variables themselves in the corresponding stratification analysis. TG/HDL, Triglyceride/High-density lipoprotein; PIR, Poverty-to-income ratio; OR, odds ratio; CI, confidence interval.

**Figure 6:**
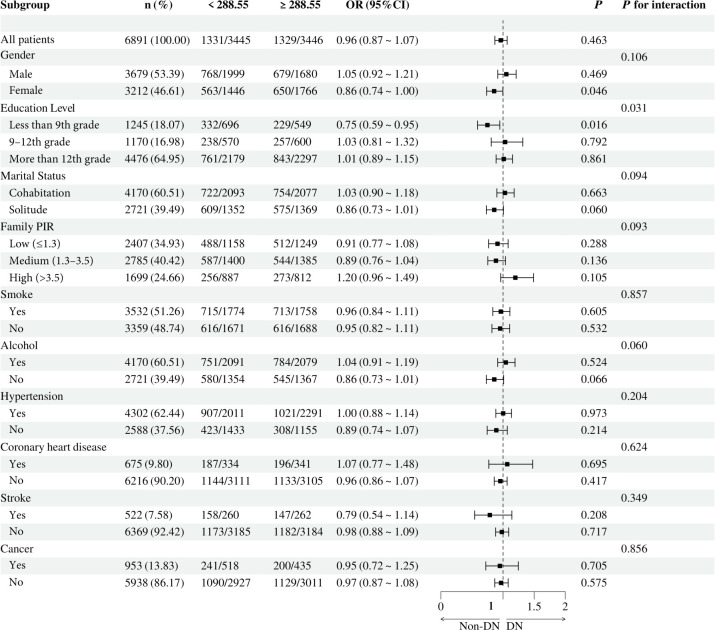
Subgroup analysis of the relationship between TyG-BMI and diabetic nephropathy. Adjusted variables: gender, age, race, education level, marital status, family PIR, smoking, alcohol, physical activity, hypertension, coronary heart disease, stroke, and cancer. The model was not adjusted for the stratification variables themselves in the corresponding stratification analysis. TyG-BMI, Triglyceride glucose - body mass index; PIR, Poverty-to-income ratio; OR, odds ratio; CI, confidence interval.

To investigate the relationship between individual indices of IR and DN in different subgroups, the analysis was stratified by gender, education, marital status, family PIR, smoking, alcohol consumption, hypertension, CHD, stroke, and cancer. The results demonstrated that, when stratified using a cut-off value of 49.98, no significant differences were observed between METS-IR levels and the incidence of DN (all *p* > 0.05). Additionally, no significant interactions were detected (all interaction *p* > 0.05), either when comparing within subgroups or examining the interaction effect across subgroups (**Figure 3**). The TyG index demonstrated a higher prevalence of DN in individuals with TyG ≥ 9.24 compared to those with TyG < 9.24 in most subgroups, except subgroups with less than 9th-grade education, confirmed CHD, and confirmed cancer. Of particular note, in the subgroup analysis of gender and smoking habits, the correlation between TyG levels and DN risk was more significant within the female subgroup and the nonsmoking subgroup. Nevertheless, no significant interaction between TyG and DN risk was observed in the other subgroups (all interaction *p* > 0.05), as illustrated in **Figure 4**. For the TG/HDL ratio, individuals with TG/HDL ≥ 3.37 exhibited a heightened risk of DN across a diverse range of subgroups, except males, individuals below the 9th grade, those belonging to different PIR subgroups, smokers, alcohol drinkers, those without hypertension, individuals with confirmed coronary artery disease, individuals with confirmed stroke, and individuals with confirmed cancer. Further analysis revealed that within the specific subgroups of education and smoking habits, the TG/HDL ratio was more strongly correlated with the risk of DN in the highly educated subgroup and the nonsmoking subgroup. No significant interaction effects were observed within the remaining subgroups (all interaction *p* > 0.05), as illustrated in **Figure 5**. Finally, in terms of the TyG-BMI index, individuals with a TyG-BMI ≥288.55 exhibited a lower prevalence of DN in the female subgroup and the subgroup up to the 9th grade compared to participants with a TyG-BMI <288.55 (all *p* < 0.05). In contrast, no significant differences were observed between TyG-BMI levels and DN prevalence in any of the remaining subgroups (all *p* > 0.05). Notably, in the subgroup analysis stratified by education, the low-education subgroup exhibited a higher correlation between TyG-BMI and DN risk. Similarly, no significant interactions were found within the remaining subgroups (all interactions *p* > 0.05), as shown in **Figure 6**.

## 4 Discussion

The objective of this study was to investigate the association between non-insulin-based IR indices (METS-IR, TyG, TG/HDL, and TyG-BMI) and DN through a cross-sectional analysis of 6,891 U.S. adults with DM from the NHANES 1999-2018 database. The findings indicated that individuals in the highest quartiles of METS-IR, TyG, TG/HDL, and TyG-BMI exhibited a markedly elevated risk of developing DN. After adjusting for multiple covariates, including gender, age, and race, this association remained significant and demonstrated a nonlinear relationship. These findings further confirm the importance of IR in the pathogenesis of DN and provide a potential assessment tool for the non-insulin-based IR indices in the prevention and management of DN.

IR is not only a core pathophysiologic feature of diabetes, but it also plays a pivotal role in the development and progression of DN (19, 37). IR contributes to the development of DN through a variety of biological pathways, including increased inflammatory response (38, 39), oxidative stress (40, 41), endothelial dysfunction (42, 43), and the promotion of accumulation of extracellular matrix (44), which collectively leads to alterations in renal structure and function. In the progression of DN, IR may contribute to glomerulosclerosis by increasing the filtration pressure in the kidney, leading to glomerular hyperfiltration (18, 45). Furthermore, IR has been linked to the dysfunction of podocytes, a crucial component of the glomerular filtration membrane (46, 47). Podocyte injury can result in the development and progression of proteinuria. Concurrently, hyperinsulinemia in the IR state may facilitate the proliferation and fibrosis of renal cells through the activation of signaling pathways, including JAK/STAT, MAPK, and PI3K/Akt (48–51).

This study revealed significant associations between all four non-insulin-based IR indices (METS-IR, TyG, TG/HDL, and TyG-BMI) and the risk of DN. This finding supports the notion that IR is a critical factor in the pathogenesis of DN. Of particular interest is that the TyG index demonstrated a highly consistent association with DN risk across all analyzed models. This result echoes several previous studies and further solidifies the utility and validity of the TyG index as a DN risk assessment tool. Several studies have confirmed the strong association between the TyG index and albuminuria (30, 52). In patients with T2DM, the TyG index was associated with DN independently of other factors, demonstrating a superior ability to identify DN compared with the traditional HOMA-IR index (29, 30). Furthermore, the METS-IR, TG/HDL, and TyG-BMI indices showed significant correlations with DN risk in the adjusted model. Notably, while all these indices of IR demonstrated potential in predicting the risk of DN, the evaluation of their predictive value varied somewhat across studies. For instance, one study in a rural Chinese population observed that a high METS-IR score was associated with an increased risk of mild decline and rapid deterioration of renal function (13). In contrast, in patients with a primary diagnosis of T2DM, the risk of DN increased with elevated TyG index and TyG-BMI. However, the efficacy in diagnosing DN was relatively low (53). Furthermore, a retrospective analysis of 521 patients with T2DM showed that among the four metrics for assessing IR, the TyG index, in conjunction with the TG/HDL ratio, exhibited the most significant predictive effect, followed by the METS-IR. In contrast, the TyG-BMI exhibited a relatively weak effect (54). The TyG index demonstrated the strongest association with DN risk in the present study, followed by the TG/HDL ratio. In contrast, the METS-IR and TyG-BMI indices exhibited relatively inferior performance. These findings reflect the differential performance of different IR indices in specific populations and emphasize the need to comprehensively consider multiple factors in clinical applications and research to develop more accurate risk assessment and intervention strategies.

Furthermore, it is essential to acknowledge that many factors, including genetic predisposition (55, 56), environmental exposures (57), lifestyle, and comorbidities (58), influence the relationship between IR and DN. The subgroup analyses conducted in this study demonstrated the impact of various demographic characteristics, lifestyle habits, and chronic disease histories on the relationship between IR and DN. For instance, the correlation between the TyG index and the risk of DN was more pronounced in the female and nonsmoking subgroups. This may be attributed to disparate patterns of insulin sensitivity or insulin secretion in women and nonsmokers (59, 60). Furthermore, the association between TyG-BMI and DN risk was more pronounced in the less educated subgroup. This may be attributed to lower socioeconomic status and health literacy, influencing patients’ lifestyle and healthcare access (61). These findings indicate that socioeconomic status, lifestyle, and personal behavior may affect the relationship between IR and DN. It is crucial to consider the specificity of different population subgroups when developing prevention and management strategies for DN.

Non-insulin-based IR indices (METS-IR, TyG, TG/HDL, and TyG-BMI) offer significant advantages over traditional methods of assessing IR (HEC and HOMA-IR) (26, 31, 33, 53). Firstly, these novel indices do not necessitate the direct measurement of insulin levels, which confers them an advantage in cost and operational complexity. The high cost of insulin or C-peptide measurements, the necessity for specific laboratory equipment and specialized personnel, and the availability of these resources in resource-limited settings limit the widespread use of these measurements in such settings. Second, non-insulin-based indices are straightforward to calculate and rely solely on routine biochemical markers, such as FPG, TG, HDL-C, and BMI, which can typically be measured in a standard clinical laboratory (62). This simplicity renders these indices more suitable for large-scale epidemiological studies and routine clinical practice. Moreover, as these indices are not dependent on insulin measurements, they are instrumental in patients with diabetes, especially those on insulin therapy. In patients receiving exogenous insulin, elevated insulin levels may not accurately reflect IR status, as the use of insulin may confound insulin sensitivity (22). Furthermore, the non-insulin-based indices’ capacity to reflect many dimensions of IR, including the severity of IR and its correlation with cardiovascular disease risk, contributes to a more comprehensive evaluation of the overall health status of diabetic patients (63–68). Finally, the practical value of these indices in predicting and assessing the risk of DN has been confirmed by previous studies and the present study. They may be advantageous in the early identification of high-risk patients, facilitating timely preventive and interventional measures.

The principal strength of this study lies in the utilization of a comprehensive, nationally representative database, NHANES, which encompasses a diverse array of population characteristics, thereby ensuring the generalizability and reliability of the findings. Second, we adjusted for confounding variables to obtain more plausible results. Furthermore, multiple indices of non-insulin-based IR were employed in this study, and detailed subgroup analyses were conducted to assess these indices’ association with DN comprehensively. Nevertheless, it should be noted that this study has limitations. First, as this was a cross-sectional study, it was impossible to determine whether the observed associations were causal. Second, although we considered several potential confounding variables, there may still be unconsidered variables, such as genetic factors and polymorphisms, which may impact the results. Future studies could further explore the impact of these factors on the association between IR and DN. Furthermore, the study was conducted primarily on a U.S. population, and the results may not be generalizable to other racial or regional groups.

## 5 Conclusion

In conclusion, the present study investigated the complex associations between non-insulin-based IR indices (METS-IR, TyG, TG/HDL, and TyG-BMI) and the risk of DN. The results demonstrated that all of these indices were significantly correlated with the risk of DN, with the most significant correlation being that of the TyG index. This finding highlights the potential application of these IR indices in the prevention and management of DN. It provides clinicians with a more accurate risk identification and management tool, which is expected to optimize the individualized treatment plan for DN patients. Future studies should further explore the application of these indices in different populations and evaluate their role in the early diagnosis and treatment of DN. In the meantime, further longitudinal studies are required to ascertain the causal relationship between these indices and DN.

## Data Availability

The datasets presented in this study can be found in online repositories. The names of the repository/repositories and accession number(s) can be found below: The National Health and Nutrition Examination Survey dataset is publicly available at the National Center for Health Statistics of the Centers for Disease Control and Prevention (https://www.cdc.gov/nchs/nhanes/index.htm).
